# Does Secondhand Smoke Affect the Development of Dental Caries in Children? A Systematic Review

**DOI:** 10.3390/ijerph8051503

**Published:** 2011-05-12

**Authors:** Takashi Hanioka, Miki Ojima, Keiko Tanaka, Mito Yamamoto

**Affiliations:** 1 Department of Preventive and Public Health Dentistry, Fukuoka Dental College, 2-15-1 Tamura, Sawara-ku, Fukuoka 814-0193, Japan; E-Mail: fujiyosi@college.fdcnet.ac.jp; 2 Department of Preventive Dentistry, Graduate School of Dentistry, Osaka University, 1-8 Yamadaoka, Suita, Osaka 565-0871, Japan; E-Mail: ojima@dent.osaka-u.ac.jp; 3 Department of Public Health, Faculty of Medicine, Fukuoka University, 8-19-1 Nanakuma, Jonan-ku, Fukuoka 814-0180, Japan; E-Mail: k-tanaka@fukuoka-u.ac.jp

**Keywords:** secondhand smoke, parental smoking, perinatal smoking, dental caries, causal assessment, motivation of smoking cessation

## Abstract

This review evaluated evidence of the relationship between secondhand smoke (SHS) and dental caries in children in epidemiological studies. Relevant literature was searched and screened, and the methodological quality was assessed. The search yielded 42 citations. High-quality studies including one cohort format and 14 case-control format studies were selected. Early childhood caries was examined in 11 studies. The independent association of SHS was significant in 10 studies, and the strength was mostly weak to moderate. One study did not select SHS as a significant variable. Three studies reported decreases in the risk of previous exposure, and the association was not significant. Dose-response relationships were evident in five studies. Permanent teeth were examined in seven studies. Five studies reported significant associations, which were mostly weak. The risk of previous exposure remained similar to that of current exposure, and a dose-response relationship was not evident in one study. The overall evidence for the causal association in early childhood caries is possible regarding epidemiological studies, and the evidence of permanent teeth and the effect of maternal smoking during pregnancy were insufficient. The results warrant further studies of deciduous teeth using a cohort format and basic studies regarding the underlying mechanism.

## 1. Introduction

The evidence for a causal association between active smoking and premature tooth loss is rapidly growing [1,2]. This association could be due to the effects of smoking on periodontal disease [3,4] and prognosis [5,6]. A plausible mechanism for the relationship between smoking and root surface caries is gingival recession due to periodontal disease [3].

Dental caries occurs predominantly in younger children, and various factors may influence development of dental caries including secondhand smoke (SHS). The basic etiology of dental caries is attributed to the interaction among a susceptible tooth surface (tooth), fermentable carbohydrates (sugar), and specific bacteria (microorganisms), in particular, *Streptococcus mutans*, which converts carbohydrates into acids. On the susceptible tooth surface, prolonged lowering of the pH in dental plaque induces demineralization of the tooth surface, resulting in the destruction of the hard structure of the tooth. Behavioral factors such as poor oral hygiene and the consumption of sugar-containing snacks are significant factors for both *S. mutans* infection and caries lesion initiation. Therefore, dental caries is a chronic, infectious, and multifactorial disease.

To the best of our knowledge, a causal association between SHS and dental caries in children has not been systematically assessed. This review focused on exploring a potential causal association between SHS and dental caries in children according to a proposal for reporting evidence of observational studies [7] and by evaluating the methodological quality of studies [8]. The primary aim of the present review is to determine if SHS affects the development of dental caries in children.

## 2. Methods

### 2.1. Literature Search

An electronic search was conducted using MEDLINE (January 1990 to September 2010) to identify pertinent literature. The search strategy applied was as follows: dental caries AND ((parental smoking) OR (maternal smoking) OR (household smoking) OR (paternal smoking) OR (secondhand smoke) OR (involuntary smoking) OR (passive smoking)). In addition, the reference lists in articles that we read completely and the latest articles, of which we were informed by a newsletter (the MDLinx, M3 Inc., Tokyo), were considered.

### 2.2. Outcome and Exposure

The primary outcome of interest was dental caries. The dmft and DMFT indices have been used most frequently for this purpose. Numbers of decayed (unfilled) tooth, missing teeth because of dental caries, and filled teeth were calculated for “d”, “m” and “f” categories for early childhood caries. The DMFT index was applied to permanent teeth. These indices are indicators of the caries experience of each individual. Therefore, prevalence based on these indices indicates the population with dental  caries experience. Because the reason for missing teeth in primary dentition is difficult to distinguish from natural replacement with permanent teeth, “m” is not used for children aged five years or older. Several variables such as serum cotinine levels, maternal smoking during pregnancy, and paternal, maternal, and household smoking can be used as a measure of SHS.

### 2.3. Eligibility Criteria and Searching Process

The inclusion criteria for studies were English language and analysis of the relationship between SHS and dental caries utilizing multivariate models. Literature reviews and basic science studies were excluded. Search results were stored using management software (iPubMedMaker 7, Sapporo, Japan) for initial screening based on the title and abstract. Two calibrated reviewers independently screened the results. Disagreements between reviewers were resolved by discussion until a consensus was reached. Final screening consisted of evaluating full-text reports, assessing studies that could evaluate the independent association.

### 2.4. Methodological Quality Assessment

We used the modified Newcastle–Ottawa Scale (NOS), which assessed each criterion for eight items regarding the methodology of observational studies [8]. One star was given when the criterion of an item was satisfied. The grouping items of NOS consist of the following three categories: selection, comparability, and ascertainment of exposure (for case-control format) or outcome (for cohort format). A maximum of two stars were given for comparable categories; one star was given if the data were adjusted for at least one traditional risk factors regarding oral health behavior, and a second star was given if a variable for socioeconomic status (SES) or dental visit was used for adjustment. A non-response rate of less than 25% and a follow-up period of one year or more along with a dropout rate of less than 25% were used for the assessment of case-control and cohort format studies, respectively. Two reviewers independently coded the items in the modified NOS. Disagreements between reviewers were resolved by discussion until a consensus was reached. Overall quality was evaluated according to the total number of stars, and studies that were given seven stars or more were considered high-quality studies.

### 2.5. Data Abstraction

The abstracted elements in the characteristics of the studies were citation and focal factor(s) regarding the association (SHS only or various factors including SHS), analytical design (case-control or cohort format), setting (number, sex, age range, country, residency, and representativeness), analytical model and factors considered for multivariate analysis, non-respondent rate for case-control format studies, observational length and follow-up rates for cohort format studies, and funding source. Subsequently, we abstracted effect size with confidence intervals and definitions of dental caries and SHS in addition to the prevalence. One reviewer abstracted these data, which were verified independently by another reviewer. Disagreements between reviewers were resolved by discussion until a consensus was reached.

### 2.6. Evaluation of Causal Association

The three elements that were extracted and used according to the Bradford Hill criteria [9] and the Surgeon General’s report [3] are as follows: strength of association, biological gradient, and natural experiment. Common descriptors for the strength of association that were defined using effect size are as follows: weak association, ≤1.49; moderate association, 1.50–2.99; and strong association, ≥3.00 [10]. The element of biological gradient was assessed by dose-response relationship. The Bradford Hill criterion of the experiment [9] was evaluated in this review by comparing the strength of association between previous and current exposure relative to non-exposure. This criterion was named as “natural experiment” in the Surgeon General’s report [3] because conducting interventional studies in which a scientist determines subjects to be removed from exposure in a manner that does not depend on any of the subject’s characteristics is difficult in humans. Although differences from current exposure should be evaluated, the reference was usually set as the non-exposure group.

Evidence concerning a causal association between SHS and dental caries in children was evaluated on the basis of the three elements by collecting evidence with regard to consistency in terms of study quality and design and by considering shortcomings of the evidence.

### 2.7. Evidence Synthesis of Consistency according to Study Quality

Results in each element were evaluated according to study quality. The following descriptors were used for interpretation of consistency [11]: strong evidence, consistent findings among multiple high-quality studies; moderate evidence, consistent findings among multiple low-quality studies and/or one high-quality study; limited evidence, one low-quality study; conflicting evidence, inconsistent findings among multiple studies; and no evidence among studies.

### 2.8. Evidence Synthesis of Association according to Study Design

Evidence synthesis was further performed by considering study design according to modification of the standardized descriptions [12]. Intervention study was replaced with natural experiment. This review excluded descriptions of biological plausibility, and the following criteria were applied:

- Convincing: Evidence is based on epidemiological studies showing consistent associations between exposure and disease, with little or no evidence to the contrary. The available evidence is based on a substantial number of studies including prospective observational studies, and where relevant, natural experiments of sufficient size, duration, and quality showing consistent effects.
- Probable: Evidence is based on epidemiological studies showing fairly consistent associations between exposure and disease, but there are perceived shortcomings in the available evidence or some evidence to the contrary, precluding a more definite judgment. Shortcomings in the evidence may be any of the following: insufficient duration of natural experiment (or studies), insufficient experiments (or studies) available, inadequate sample sizes, and incomplete follow-up.
- Possible: Evidence is mainly based on the findings of case-control and cohort formats studies. Insufficient numbers of natural experiments or observational studies are available. Additional natural experiments are required to support the tentative associations.
- Insufficient: Evidence is based on the findings of a few studies that are suggestive but insufficient to establish an association between exposure and disease. Limited or no evidence is available from natural experiments. Additional well-designed research is required to support the tentative associations.

## 3. Results and Discussion

### 3.1. Number of Studies

**Figure 1.**
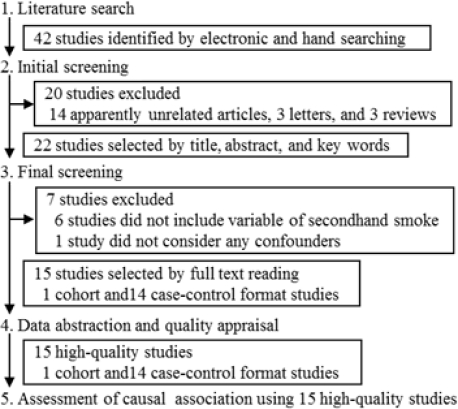
Number of studies according to the processes of searching, selection, and evaluation of literature.

The electronic and hand searches yielded 42 citations (Figure 1). The initial screening identified 22 relevant studies. The remaining studies that included apparently unrelated articles, letters and review articles were excluded from the review. Based on a full-text review of the literature, we selected 15 studies [13–27] and excluded seven studies (citations not shown).

### 3.2. Characteristics of Studies

Fourteen studies used a case-control format [13–24,26,27], and one study used a cohort format [25] (Table 1). Nine studies focused on SHS [14–16,18,20,21,23,24,26]. Another study focused on social variables including SHS [13,22], low birth weight [17], breastfeeding [19], and associated factors [25,27]. Studies were conducted in the United States (US) [15–17,19,27], Japan [18,21,22,24,26], eight European countries [13], the United Kingdom (UK) [14], South Africa [20], Belgium [23], and Sweden [25]. Data were obtained from national records [14,15,17–19,25], and records in  schools [13,20,23,26,27], public health facilities [21,22,24] and hospitals [16]. Variables used for adjustment were demographic factors, oral health behavior including dental visit, SES, and other possible confounders. The non-respondent rate was not reported in six studies [15–17,19,20,27]. Sources of funding were derived from public health organizations [13–16,18–21,24–27] and oral health companies [13,23]. Two studies did not describe the funding source [17,22].

### 3.3. Quality of Studies

According to the modified NOS, all studies were classified as high-quality studies (Table 2). Nine and six studies did not receive any star because of the lack of a reported non-response rate and comparability, respectively. These items should be carefully considered in future studies.

### 3.4. Strength of Association

Among 15 high-quality studies, 11 studies reported relationships between SHS exposure and early childhood caries (Table 3). All studies used a case-control format. Significant associations were reported in 10 studies. In one study, SHS was not selected as a significant variable in the forward stepwise regression analysis [17]. Strong associations were reported in two studies [16,23], moderate associations were reported in five studies [13,14,19,21,22], and weak and moderate associations were reported in two studies [15,24]. Weak association was noted in one study that reported a high prevalence of dental caries (78.5%) [26]. The strength of association may be diluted in populations with a high prevalence of dental caries. The prevalence of dental caries was over 20%, and thus odds ratios may overestimate the relative risk. Studies that reported strong association showed wide confidence interval, which indicates a low precision of strong association. Therefore, modest description of the association was weak to moderate. The association with maternal smoking during pregnancy was moderate in two studies [19,24], and no significant association was found in one study (effect size was not available) [17].

The association of SHS with caries of permanent teeth was examined in seven studies (Table 4).

Two studies combined deciduous and permanent teeth in the model [18,26]. Among five studies that reported significant associations [13,20,25–27], one study used a cohort format and examined the association with maternal smoking during pregnancy [25]. Weak associations were reported in four studies [13,25–27], and one study reported a moderate association [20]. The moderate association may be a result of the limitation of specific teeth that could compromise the effects of confounders. In general, the association of SHS with caries of permanent teeth was weak.

### 3.5. Natural Experiment

Effect size between previous and current exposure was compared in Japan [24,26] and Belgium [23] (Table 5). The risk of childhood dental caries due to previous exposure was not significant, while the risk due to current exposure was significant. Decreased risk was apparent in one study [23], while two other studies reported smaller changes [24,26] including a study that examined maternal smoking during pregnancy [24]. For permanent teeth, the risk in both exposure groups was identical [26]. Evidence for consistent trend of decreased risk of early childhood caries was limited. Further studies of early childhood caries should be conducted in various populations to further clarify the trend of this element.

### 3.6. Dose-Response Relationship

Dose-response relationships were examined in Japan [21,22,24,26] and the US [15] (Table 6). Three studies compared the relationship by three levels of exposure [21,22,24], and two studies employed four levels [15,26]. Positive associations were evident in all studies. Effect sizes of the two highest levels of exposure were similar. It is likely that there is a threshold in the increase of risk by SHS. The reason should be clarified, for example, sensitivity of developing dental caries against exposure to SHS. Similar to the results of natural experiment, the trend of the dose-response relationship was apparent for early childhood caries. Only one study examined the dose-response relationship in permanent teeth, but the relationship was not clearly outlined [26]. Additional studies in various populations and for permanent teeth are needed for definitive assessment of this element.

### 3.7. Evaluation of Evidence in Epidemiological Studies

Evidence for each element in high-quality studies was summarized by tooth type (Table 7). The strength of association was weak to moderate for primary dentition. Overall evidence with respect to consistency was moderate to strong for three elements. However, natural experiment and dose-response relationship were reported from limited countries. Apart from biological plausibility, the epidemiological evidence regarding the causal association of SHS exposure with early childhood caries is possible. Although epidemiological evidence for weak associations in permanent dentition has recently increased, evidence for natural experiment and the dose-response relationship is very limited. Therefore, evidence for caries in permanent teeth is insufficient for evaluating the causal relationship.

The impact of maternal smoking during pregnancy would be similar to that of household smoking by parents; however, limited data is available to substantiate this hypothesis. The effect was examined in three studies for early childhood caries with a case-control format [17,19,24], and the association was significant in two studies [19,24]. Another study of permanent teeth with a cohort format reported a significant association [25]. One study demonstrated a decreased risk of early childhood caries due to previous exposure [24], but a dose-response relationship was not reported. The association of SHS was more evident with primary teeth than with permanent teeth. Interactions between exposure to SHS and timing of mineralization before eruption and etiologic factors after eruption may explain the observed difference. Epidemiological studies regarding the effect of maternal smoking during pregnancy on dental caries in children should be further conducted to elucidate the relationship.

The results of studies for early childhood caries with a case-control format may be comparable to those of cohort studies. Indices represented caries experience following eruption of teeth. All records collected in cross-sectional format survey were generally used in the statistical analysis of the case-control format. Therefore, all cases of dental caries following tooth eruption and during SHS exposure could be analyzed by the model without exception. Although the history of confounders  following eruption of teeth should be carefully considered for this interpretation, the description of evidence based on studies with a case-control format may be conservative.

Although each study considered various factors, no study could include all variables completely that are related to dental caries in children. Therefore, the significant association between SHS and dental caries observed in each study may miss a possible effect of confounder that was not entered in the model. In the present review, this kind of error may be less because the common confounder that was not entered in multivariable models was not observed (Table 1).

This study was performed according to standard criteria of the Surgeon General’s Report [3] that did not employ meta-analysis for assessment of causal association, though the assessment included articles that used meta-analysis. In the present review, article that used meta-analysis was not identified. Application of quantitative meta-analysis including heterogeneity analysis could further clarify strength of association.

### 3.8. Biological Plausibility

The most straightforward explanation of the relationship between SHS and dental caries is social inequality. Enamel defects were associated with development of early childhood caries in a population with low SES [28]. To the best of our knowledge, association between SHS and dental caries was first reported using SHS as a marker of low SES [13]. This factor is also the strongest determinant of oral health inequality [29]. SHS may be a marker for unhealthy choices in diet and oral hygiene practices.  However, the association between SHS and childhood dental caries was still significant after adjustment for variables of SES and other related factors.

**Figure 2.**
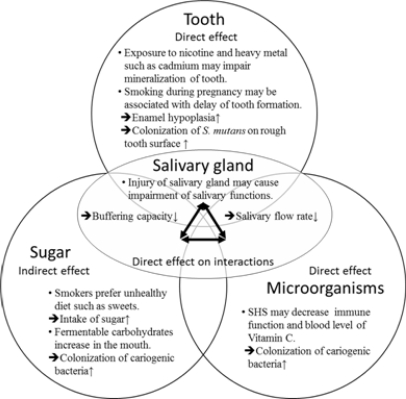
Summary of the biological mechanism of SHS directly or indirectly affecting dental caries in children according to the traditional explanation of the etiology of dental caries.

The underlying mechanism could be explained as a modulation of existing etiology of dental caries. In the reviewed literature of epidemiological studies, several pathways were proposed for biological plausibility. These pathways were summarized on the basis of the traditional etiology of dental caries (Figure 2). SHS may directly influence tooth and oral microorganisms. Exposure to SHS during the period of tooth formation may influence mineralization [30–33]. Environmental cadmium exposure may be independently associated with increased risk of early childhood caries [34]. Colonization of cariogenic bacteria on rough tooth surfaces could be enhanced by SHS. The number of cariogenic microorganisms may increase with a decrease in immune function. Exposure to SHS may predispose subjects to infections through suppression or modulation of the immune system [35]. Blood levels of vitamin C in smokers and children who reside with smoking parents were decreased [36–38]. Decreased vitamin C levels were associated with the growth of *S. mutans* [39]. The influence of sugar would be an indirect and apparent relationship because of unhealthy lifestyles of smoking parents [40,41]. This effect, if any, may not be reflected in the effect size of risk estimate because of adjustment for this variable.

Another notable factor in addition to the traditional etiologic factors is the salivary gland, which may be more definitive than the previously mentioned explanation. Saliva contains various biologically active components and affects basic factors of dental caries etiology. The buffering capacity of saliva decreases and the numbers of lactobacilli and *S. mutans* in saliva increases in smokers [42] and children who reside with smokers [43]. Low levels of saliva because of low salivary gland function [44] may also enhance the colonization of cariogenic bacteria on rough tooth surfaces and interrupt clearance of fermentable carbohydrate from the mouth. Decreased function of the salivary gland may also influence remineralization of tooth surfaces.

The tentative effects of SHS regarding tooth formation and salivary gland function would be altered during pregnancy as well as after delivery. The present review did not find sufficient evidence regarding maternal smoking during pregnancy. However, results of maternal smoking as a measure of SHS included maternal smoking during pregnancy in most studies. Further studies that focus on maternal smoking during pregnancy should be conducted to examine these hypotheses.

Another factor relevant to SHS could modulate the basic etiology and may further increase susceptibility to dental carries. Children may inhale SHS through the mouth because of nasal congestion [45], and breastfeeding from a mother who smokes may result in toxic substances being delivered to the child’s mouth [46]. Tobacco smoking was associated with elevated levels of *S. mutans* and lactobacilli in saliva [47]. These findings may indicate increased susceptibility to dental caries in children who reside with smoking parents, possibly resulting in the early colonization of *S. mutans* [48,49].

## 4. Conclusions

Recent studies on the association of SHS with early childhood caries exhibited potential to infer a causal association. The available evidence in high-quality epidemiological studies revealed an independent association by adjustments for low SES and other related factors. Evidence of a causal association between SHS and early childhood caries is possible by the assessments based on standardized elements of evaluation excluding biological plausibility. Further studies should be conducted to examine whether SHS is a true risk of early childhood caries by employing unmeasured covariates in the past studies. For example, including a variable of salivary function might validate biological plausibility. The results warrant further studies using a cohort format. The relationship between maternal smoking during pregnancy and permanent dentition, and the underlying mechanism should be further clarified.

## Figures and Tables

**Table 1.** Characteristics of the included studies

**Study and focal factor**	**Setting**	**Statistical method and non-respondent rate**	**Funding**
Bolin, 1997 [13], SES ^a^	CS ^b^ data of school children in 8 European countries	Logistic RA ^c^ adjusted for country, type of household, number of siblings, parent’s age and social class; non-respondent rate of 4.6%	PHOs ^d^, OHCs ^e^
Williams, 2000 [14], SHS	CS ^b^ data from the UK NDNS^f^ (1995)	Logistic RA ^c^ adjusted for age ^g^, gender, social class ^g^, household expenditure on confectionery ^g^, and height; non-respondent rate of 23%	PHO ^d^
Aligne, 2003 [15], SHS	CS ^b^ data from the NHANES^h^ III (1988–1994) in the US	Logistic RA ^c, i^ adjusted for age, region of residence, race/ethnicity, time since last dental visit, poverty status and education level, non-respondent rate ND ^j^	PHOs ^d^
Shenkin, 2004 [16], SHS	CS ^b^ data from IFS ^k^ in 8 hospitals in the US	Logistic RA ^c^ adjusted for age, toothbrushing frequency, total infested fluoride, and SES ^b^; non-respondent rate ND ^j^	PHOs ^d^
Shulman, 2005 [17], low birth weight	CS ^b^ data from the NHANES^h^ III (1988–1994) in the US	Poisson RA ^c^ adjusted for age, race/ethnicity, income, history of breast-feeding, and bottle use after 19 months; non-respondent rate ND ^j^	ND ^j^
Tanaka, 2006 [18], SHS	CS ^b^ data from the NNS^l^, NSDD ^m^ (1999) in Japan	Logistic RA ^c^ adjusted for age, sex, region of residence, tooth brushing frequency, experience of topical fluoride application, and body mass index; non-respondent rate of 45.9% of participants of the NNS ^l^	PHOs ^d^
Iida, 2007 [19], infant breastfeeding	CS ^b^ data from the NHANES ^h^ (1999–2002) in the US	Logistic RA ^c^ and Poisson RA ^c^ adjusted for age, race/ethnicity, time since last dental visit, poverty status, history of breast feeding, birth weight, and maternal age; non-respondent rate ND ^j^	PHOs ^d^
Ayo-Yusuf, 2007 [20], SHS	CS ^b^ data in 21 high schools in South Africa	Logistic RA ^c^ adjusted for age, sex, ethnicity/race, family structure, sugar intake frequency, daily brushing frequency, time since dental visit, income of breadwinner, and current smoking status of the respondent; non-respondent rate ND ^j^	PHOs ^d^
Hanioka, 2008 [21], SHS	CS ^b^ data from records of health checkup at a public health facility in Japan	Logistic RA ^c^ adjusted for order of birth, types of main drink, frequency of daily intake of sugar-containing snacks, daily toothbrushing by parents, use of fluoridated toothpaste, and residential location; non-respondent rate of 15.9%	PHOs ^d^
Aida, 2008 [22], social context to inequality	CS ^b^ data from records of health checkup at facilities in 39 municipalities in Japan	Multilevel RA ^c^ adjusted for age, order of birth, living with grandparents, toothbrushing-related habits, use of fluoride paste, taking sweet foods and drinks, occupation of household and termination of breast feeding, and 9 community-level variables; non-respondent rate of 20.1% for municipalities and 6.5% for participants of the participated municipalities	ND ^j^
Leroy, 2008 [23], SHS	CS ^b^ data from the Smile for Life project in 4 distinct geographical areas in Flanders, Belgium	Logistic RA ^c^ adjusted for sex, home situation, dental plaque, age at start brushing, help with brushing, brushing frequency, use of nursing bottle, application of sweetener on a pacifier, cleaning a pacifier in the own mouth and in between meals/drinks, drinks/snacks at night, and educational level; non-respondent rate of 17.0% and 14.8% of children aged three and five years, respectively	OHCs ^e^
Tanaka, 2009 [24], SHS	CS ^b^ data from the FCHS^n^ in 7 public health centers in Japan	Binomial RA ^c^ with log link function adjusted for sex, toothbrushing frequency, use of fluoride, between-meal snack frequency, and paternal and maternal educational level; non-respondent rate of 74.5%	PHOs ^d^
Julihn, 2009 [25], associated factors	Cohort data from the SMBR^o^ and SNR ^p^ available at Statistics Sweden	Logistic RA ^c, g^ adjusted for child’s country of birth, sex, maternal age, parental country of birth, education level, marital status, mother receiving social welfare allowance, and the interaction term “unmarried mothers” and “mother receiving social welfare allowance;” non-respondent rate of 14%	PHOs ^d^
Tanaka, 2010 [26], SHS	CS ^b^ data from school records in Okinawa, Japan	Binomial RA ^c^ with the log link function adjusted for age, sex, region of residence, toothbrushing frequency, use of fluoride, sugar intake, and paternal and maternal educational level; non-respondent rate of 24.4%	PHOs ^d^
Ditmyer, 2010 [27], associated factors	CS ^b^ data in schools in Nevada	Logistic RA ^c^ adjusted for age, sex, race, living in an area with or without community water fluoridation, applied dental sealants, dental insurance status, and smoking habits; non-respondent rate ND ^j^	PHOs ^d^

a SES,

b cross-sectional,

c regression analysis,

d public health organization,

e oral health company,

f National Diet and Nutrition Survey,

g variables entered in the final model,

h National Health and Nutrition Examination Survey,

i adjusted for the statistically significant variables in another model,

j not described,

k Iowa Fluoride Study,

l National Nutrition Survey,

m National Survey of Dental Diseases,

n Fukuoka Child Health Study,

o Swedish Medical Birth Register,

p Swedish National Registers.

**Table 2.** Methodological quality assessed using the modified NOS.

**Study**	**Selection**				**Comparability**		**Exposure^a^/Outcome^b^**			**Total stars**
	**1 ^c^**	**2 ^c^**	**3 ^c^**	**4 ^c^**	**5 ^d^**		**6 ^c^**	**7 ^c^**	**8 ^c^**	
Bolin, 1997 [13]	*	*	*	*		*	*	*	*	8
Williams, 2000 [14]	*	*	*	*	*	*	*	*	*	9
Aligne, 2003 [15]	*	*	*	*	*	*	*	*		8
Shenkin, 2004 [16]	*		*	*	*	*	*	*		7
Shulman, 2005 [17]	*	*	*	*		*	*	*		7
Tanaka, 2006 [18]	*	*	*	*	*		*	*		7
Iida, 2007 [19]	*	*	*	*		*	*	*		7
Ayo-Yusuf, 2007 [20]	*	*	*	*	*	*		*		7
Hanioka, 2008 [21]	*	*	*	*	*		*	*	*	8
Aida, 2008 [22]	*	*	*	*	*	*	*	*		8
Leroy, 2008 [23]	*	*	*	*	*	*	*	*	*	9
Tanaka, 2009 [24]	*		*	*	*	*	*	*		7
Julihn, 2009 [25]	*	*		*		*	*	*	*	7
Tanaka, 2010 [26]	*	*	*	*	*	*	*	*	*	9
Ditmyer, 2010 [27]	*	*	*	*		*	*	*		7

a case-control format,

b cohort format,

c for studies with case-control/cohort format: 1, definition of case/representativeness of exposed cohort; 2, representativeness of case/selection of non-exposed cohort; 3, selection of controls/ascertainment of exposure; 4, definition of controls/demonstration that outcome of interest was not present at the start of the study; 6, ascertainment of exposure/assessment of outcome; 7, same method for cases and controls/follow-up period of one year or more, 8, non-response rate of less than 25%/adequacy of follow up of cohorts;

d 5, study controls for oral health behavior and SES or dental visit.

**Table 3.** Relationship between SHS exposure and caries of deciduous teeth sorted by age of children.

**Study**	**Age (n)**	**SHS**		**Dental caries**		**Effect size OR ^c^ (95% CI ^d^)**
		**Def ^a^**	**%**	**Def ^b^**	**%**	
Leroy, 2008 [23]	3 (1,038)	HS	30.9	dmft	6.7	1.98 (0.68, 5.76)
	5 (1,093)	HS	30.2	dmft	30.2	3.36 (1.49, 7.58)
Aida, 2008 [22]	3 (3,086)	PS, MS	20.4	dmft	31.3 ^e^	2.14 (1.59, 2.87) ^e^
Hanioka, 2008 [21]	3 (711)	PS, MS	34.5	dmft	35.7	2.25 (1.51, 3.37)
Tanaka, 2009 [24]	3 (2,015)	MSP only	2.3	dmft	20.8	1.78 (1.16, 2.75) ^f^
		HS only	31.8	dmft	20.8	1.26 (1.04, 1.53) ^f^
		Both	10.7	dmft	20.8	1.40 (1.08, 1.81) ^f^
Iida, 2007 [19]	2–5 (1,563)	MSP	14.5	dfs	27.2	1.68 (1.01, 2.79)
Williams, 2000 [14]	3–4.5 (729)	MS	33	dmft	25.2	1.54 (1.07, 2.21)
Shulman, 2005 [17]	2–6 (4,207)	MSP	5.2	dfs count	NA	Not significant ^g^
Bolin, 1997 [13]	5 (1,423)	MS	36.8 ^e^	dmfs	46.9 ^e^	1.52 (1.18, 1.95) ^e^
Shenkin, 2004 [16]	4–7 (637)	HS	10.2	dfs	26.2 ^e^	3.38 (1.68, 6.79)
Aligne, 2003 [15]	4–11 (3,531)	SCL	55.4	ds	25.7	1.8 (1.2, 2.7)
				Fs	33.2	1.4 (1.1, 2.0)
Tanaka, 2010 [26]	6–15 (13,863)	HS ^h^	15.9	dft	78.5	1.06 (1.04, 1.08) ^f^

a definition of secondhand smoke: HS, household smoking; PS, paternal smoking; MS, maternal smoking; MSP, maternal smoking during pregnancy; SCL, serum cotinine level;

b definition of dental caries: t, tooth; s, surface;

c odds ratio;

d confidence interval;

e calculated by reviewer based on data in the table in the original literature;

f prevalence ratio;

g incidence density ratio;

h household smoking ≥15 cigarettes daily.

**Table 4.** Relationship between SHS exposure and caries of permanent teeth sorted by age of children.

**Study**	**Age (n)**	**SHS**		**Dental caries**		**Effect size OR ^c^ (95% CI ^d^)**
		**Def ^a^**	**%**	**Def ^b^**	**%**	
Tanaka, 2006 [18]	1–14 (925)	HS	42.5	dft, DFT	61.2	1.26 (0.93, 1.69)
Aligne, 2003 [15]	4–11 (2,930)	SCL	54.2	DS	9.0	1.2 (0.8, 1.9)
				FS	18.9	0.9 (0.6, 1.3)
Tanaka, 2010 [26]	6–15 (20,253)	HS^e^	16.5	DFT	55.1	1.03 (1.00, 1.06) ^f^
	6–15 (20,703)			dft, DFT	83.0	1.04 (1.03, 1.06) ^f^
Bolin, 1997 [13]	12 (1,265)	MS	31.4 ^g^	DMFS	62.8 ^g^	1.40 (1.11, 1.78) ^g^
Ayo-Yusuf, 2007 [20]	14.6 ^h^ (1,873)	HS	36.9	DT ^i^	16.4	2.02 (1.22, 3.33)
Julihn, 2009 [25]	13, 19 ^j^ (15,538)	MSP	21.0	ACI ^k^	38.6	1.33 (1.22, 1.44)
Ditmyer, 2010 [27]	12–19 (4,169)	HS	34.6	DMFT	NA ^l^	1.42 (1.03, 1.53)

a definition of secondhand smoke: SCL, serum cotinine level; HS, household smoking; MS, maternal smoking; MSP, maternal smoking during pregnancy;

b definition of dental caries: T, tooth; S, surface;

c odds ratio;

d confidence interval;

e household smoking ≥15 cigarettes daily;

f prevalence ratio;

g calculated by reviewer based on data in the table in the original literature;

h average of high school students;

i decayed second molar;

j cohort format between two ages;

k approximal caries increment;

l not available because of exclusion of middle group regarding severity of dental caries (4.0 > DMFT > 0).

**Table 5.** Effect of previous exposure to SHS on dental caries.

**Study**	**Age**	**SHS ^a^**	**Dental caries ^b^**	**Effect size (95% CI ^d^)**		
				**Def ^c^**	**SHS exposure**	
					**Former**	**Current**
Leroy, 2008 [23]	3	HS	dmft	OR	1.71 (0.30, 9.65)	1.98 (0.68, 5.76)
	5	HS	dmft	OR	0.55 (0.19, 1.65)	3.36 (1.49, 7.58)
Tanaka, 2009 [24]	3	MSP	dmft	PR	1.21 (0.91, 1.59)	1.43 (1.07, 1.91)
		HS	dmft	PR	1.23 (0.88, 1.71)	1.25 (1.04, 1.50)
Tanaka, 2010 [26]	6–15	HS	dft	PR	1.02 (0.99, 1.06)	1.06 (1.04, 1.08)
		HS	DFT	PR	1.03 (1.00, 1.06)	1.03 (1.00, 1.06)

a definition of SHS: HS, household smoking; MSP, maternal smoking during pregnancy;

b definition of dental caries;

c OR, odds ratio; PR, prevalence ratio;

d confidence interval.

**Table 6.** Dose-response relationships between SHS exposure and dental caries.

**Study**	**Age**	**SHS ^a^/Dental caries ^b^**	**Level of exposure/Effect size**			
			**1**	**2**	**3**	**4**
Hanioka,	3	PS	None	Father only	Both parents	
2008 [21]		dmft	1.00 ^c^	1.52 (1.01, 2.30) ^c^	2.25 (1.51, 3.37) ^c^	
Aida,	3	PS	None	Either parent	Both parents	
2008 [22]		dmft	1.00 ^c^	1.40 (1.15, 1.71) ^c, d^	2.14 (1.59, 2.87) ^c, d^	
Tanaka,	3	MS	None	0.1–17.9 ^e^	18.0– ^e^	
2009 [24]		dmft	1.00 ^f^	1.16 (0.93, 1.44) ^f^	1.33 (1.09, 1.63) ^f^	
Aligne,	4–11	SCL	<0.05 ^g^	0.05–<0.2 ^g^	0.2–1.0 ^g^	>1.0 ^g^
2003 [15]		ds	1.0 ^c^	1.3 (0.8, 2.3) ^c^	2.2 (1.3, 3.6) ^c^	2.3 (1.4, 3.9) ^c^
		fs	1.0 ^c^	1.1 (0.7, 1.8) ^c^	1.6 (1.0, 2.4) ^c^	1.5 (1.0, 2.3) ^c^
Tanaka,	6–15	HS	None	0.1–2.9 ^h^	3.0–6.9 ^h^	≥7.0 ^h^
2010 [26]		dft	1.00 ^e^	1.04 (1.00, 1.09) ^f^	1.12 (1.07, 1.17) ^f^	1.11 (1.06, 1.16) ^f^
		DFT	1.00 ^e^	1.12 (1.06, 1.20) ^e^	1.18 (1.12, 1.26) ^e^	1.17 (1.11, 1.24) ^e^

a definition of SHS: PS, parental smoking; MS, maternal smoking; SCL, serum cotinine level; HS, household smoking;

b definition of dental caries;

c odds ratio;

d calculated by reviewer based on data in the table in the original literature;

e pack-months;

f prevalence ratio;

g ng/mL;

h pack-years.

**Table 7.** Summary of results to evaluate the causal association between SHS and dental caries.

**Tooth type**	**Element**	**Description for consistency (number of study)**	**Evidence synthesis according to study quality**	**Perceived shortcoming**	**Evidence synthesis**
Deciduous tooth	Strength of association	Strong (2), moderate (5), weak and moderate (2), weak (1), no association (1)	Moderate to strong evidence for weak to moderate association	There was no control group in current smokers in natural experiment.	Possible evidence
	Natural experiment	Smaller effect size and no association (3) in previous exposure group	Strong evidence for natural experiment	Natural experiment and dose-response relationship were reported from two countries. Cohort study was not conducted.	
	Dose-response relationship	Obvious relationship between level of exposure and the effect size (5)	Strong evidence for dose-response relationship.		

Permanent tooth	Strength of association	Moderate (1), weak (5 ^a^), and no association (2)	Moderate evidence for weak association	Only one study reported results of natural experiment and dose-response relationship.	Insufficient evidence
	Natural experiment	Effect size was similar in previous and current exposure groups (1)	Limited evidence for natural experiment		
	Dose-response relationship	Relationship between level of exposure and the effect size was not clear (1)	Limited evidence for natural experiment		

a one study with cohort format.
